# Health care providers’ attitudes toward and experiences delivering oral PrEP to adolescent girls and young women in Kenya, South Africa, and Zimbabwe

**DOI:** 10.1186/s12913-021-06978-0

**Published:** 2021-10-18

**Authors:** Michele Lanham, Kathleen Ridgeway, Maryline Mireku, Definate Nhamo, Diantha Pillay, Mercy Murire, Kayla Stankevitz, Jordan Kyongo, Saiqa Mullick, on behalf of the OPTIONS Consortium Research Team, Taurai Bhatasara, Lina Digolo, Theresa Hoke, Annrita Ikahu, Patriciah Jeckonia, Jordan Kyongo, Michele Lanham, Megan Lydon, Nicole Makahamadze, Maryline Mireku, Wanjiru Mukoma, Saiqa Mullick, Mercy Murire, Joseph Murungu, Getrude Ncube, Definite Nhamo, Christian Ochieng, Diantha Pillay, Subarna Pradhan, Kathleen Ridgeway, Katie Schwartz, Patience Shamu, Kayla Stankevitz

**Affiliations:** 1 FHI 360, 359 Blackwell Street, Suite 201, NC 27707 Durham, USA; 2 LVCT Health, P.O. Box 19835-00202 KNH, Argwings Kodhek Road, Nairobi, Kenya; 3 Pangaea Zimbabwe AIDS Trust, 27 Rowland Square, Milton Park, Harare, Zimbabwe; 4 Wits Reproductive Health and HIV Institute, 31 Princess of Wales Terrace, 2193 Parktown, Johannesburg, South Africa

**Keywords:** HIV, Women, Adolescent, Attitude, Africa, Health care providers

## Abstract

**Background:**

In Kenya, South Africa, and Zimbabwe, oral pre-exposure prophylaxis (PrEP) is recommended for adolescent girls and young women (AGYW) at high risk of HIV. Health providers play a critical role in the uptake and effective use of sexual and reproductive health services; however, few published studies have explored providers’ attitudes toward and experiences delivering PrEP to AGYW.

**Methods:**

We conducted a cross-sectional qualitative study, interviewing 113 providers at 36 public, private, and nongovernmental health facilities in Kenya, South Africa, and Zimbabwe that were offering PrEP during the research period or were likely to offer PrEP in the future. Data were coded in NVivo 11, and an applied thematic analysis was conducted.

**Results:**

Most providers preferred that adolescent girls wait until age 18 to have sex but acknowledged that many girls younger than 18 could benefit from oral PrEP. Their primary concern was whether adolescent girls would be able to take PrEP daily, especially if they do not tell their parents or partners they are using it. Providers reported that it was more challenging to deliver PrEP and other HIV services to girls younger than 18. Those with experience providing PrEP pointed to stigma and lack of PrEP awareness in communities as two primary barriers to PrEP uptake and use.

**Conclusions:**

Providers were generally accepting of oral PrEP as an HIV prevention option for AGYW; however, many had negative attitudes about adolescent girls being sexually active and concerns about whether they could take PrEP daily. Results were used to update national PrEP training materials to address negative provider attitudes about PrEP use by AGYW.

**Supplementary Information:**

The online version contains supplementary material available at 10.1186/s12913-021-06978-0.

## Background

Adolescent girls and young women (AGYW) in eastern and southern Africa are disproportionately affected by HIV. In 2016, AGYW accounted for 26 % of the regional HIV incidence, despite being only 10 % of the population [1]. Existing HIV prevention options — including male and female condoms, abstinence, limiting the number of sexual partners, knowing your partner’s HIV status, and antiretroviral therapy (ART) for HIV-positive partners — can be difficult for an adolescent girl or young woman to use because they require the cooperation of her male partner and prevailing gender norms hinder AGYW’s decision-making power and agency in relationships [2–7].

Oral pre-exposure prophylaxis (hereafter referred to as “PrEP”) has the potential to promote choice and control over HIV prevention because it can be used without a partner’s knowledge or consent [8–10]. In 2015, WHO recommended daily PrEP — in the form of a tablet containing emtricitabine/tenofovir disoproxil fumarate — for use by individuals at ongoing substantial risk of HIV [11]; by June 2021, it was being offered in 84 countries [12]. In Kenya, South Africa, Zimbabwe, and other countries in southern and eastern Africa, PrEP is recommended for high-risk AGYW subpopulations, such as those who engage in transactional and intergenerational sex, experience gender-based violence (GBV), have sexually transmitted infections (STIs), are married or have multiple sexual partners, have partner(s) of unknown HIV status, or have older sexual partners, as well as those who perceive themselves to be at risk of HIV acquisition [13–17]. PrEP can effectively protect AGYW from HIV if they are able to access and use it as instructed [18–21].

Health providers play a critical role in the uptake and effective use of sexual and reproductive health services [22–26]. Studies among health providers suggest that their knowledge, attitudes, and beliefs about PrEP and potential PrEP clients influence their willingness to offer it [27–30]; however, few published studies explore providers’ attitudes toward [31, 32] and experiences with delivering PrEP to AGYW. A better understanding of providers’ perceptions of PrEP use by AGYW is needed to identify potential barriers to delivery of PrEP to AGYW. In addition, the experiences of providers who are already delivering PrEP to AGYW can provide insights into barriers to PrEP uptake and use among AGYW and strategies for addressing those barriers.

The Optimizing Prevention Technology Introduction On Schedule (OPTIONS) Consortium is a project that provided technical assistance from 2015 to 2020 to countries rolling out oral PrEP, with a focus on AGYW. In Kenya, South Africa, and Zimbabwe, the OPTIONS Consortium provided support to national- and county-level PrEP technical working groups, which included national health officials and other key stakeholders responsible for introducing and scaling up PrEP. Among other activities, OPTIONS conducted surveys and follow-up in-depth interviews in 2017‒2018 with providers in the three countries — both those experienced in PrEP delivery (“PrEP-experienced”) and those who had not delivered PrEP (“PrEP-inexperienced”) — to understand providers’ attitudes toward and experiences with PrEP delivery to adolescent girls (ages 15 to 17) and young women (18 to 24).^1^ Conducted in the early phases of PrEP rollout at the request of the PrEP technical working groups, this research was designed to be responsive to individual country needs, ultimately informing provider training and PrEP service delivery to AGYW in each country. The provider perspectives summarized in this paper identify attitudes and experiences that could also inform PrEP rollout to AGYW in other countries in sub-Saharan Africa.

## Methods

### Study setting

South Africa began PrEP rollout in 2016 using a phased approach that first targeted sex workers and then expanded to include men who have sex with men (MSM) in May 2017 and AGYW in October 2017. Zimbabwe also implemented a phased approach, starting in 2015 with nongovernmental organization (NGO) clinics, and then expanding to central, district, and clinic levels, respectively; AGYW were included as a priority population for PrEP from the beginning of rollout. Kenya took a different approach, rolling out PrEP nationally starting in 2017 and targeting a number of at-risk groups, including AGYW.

### Study design

This qualitative study was conducted as part of a sequential explanatory design. An initial quantitative survey was conducted with 609 providers at 60 facilities to assess providers’ attitudes about PrEP for AGYW. The survey showed that some providers held attitudes indicating reservations about providing PrEP to AGYW (see Additional File 1) [33–35]. The qualitative study was used to interpret and clarify the results from the quantitative survey. Specifically, we sought to understand what providers’ primary concerns were so they could be addressed through training. In addition, we conducted in-depth interviews with providers at 36 public, private, and nongovernmental health facilities that were offering PrEP during the research period or were likely to offer PrEP in the future.

### Sampling

The survey responses were used to inform sampling for the qualitative study. One author (KR) created a continuous score based on responses to the attitudinal questions in the survey described above to inform sampling for the qualitative component. Questions had five-point Likert scale response options ranging from 1, “Strongly Agree,“ to 5, “Strongly Disagree.“ Negatively framed questions, such as, “It’s better to tell sexually active unmarried AGYW to abstain from sex rather than give her PrEP,“ were reverse-scored before creating the summary measure. The continuous score had a minimum value of 20 and maximum of 100, with higher values indicating more positive attitudes. Survey participants with the highest and lowest scores in each country were selected for in-depth interview (IDI) recruitment (extreme case sampling) [36]. We used this sampling approach to ensure we understood the extremes of providers’ attitudes about PrEP for AGYW. Additional IDI participants with high and low attitude scores were selected as needed to ensure representation of cadres, provinces/districts, facility types (public/private/nongovernmental and primary/secondary/tertiary), and sex (see Table 1).

**Table 1:** Summary of study site and participant selection by country

	Kenya	South Africa	Zimbabwe
Region selection	Four counties were selected based on high HIV incidence and rural/urban mix: Homabay, Kisumu, Kitui, and Nairobi.	*PrEP experienced*: Four provinces included: Limpopo, KwaZulu-Natal, Gauteng, and Western Cape. Selection was based on facility rather than region (see below). *PrEP inexperienced*: Three sub-districts — located in the provinces of Gauteng, Eastern Cape, and Free State — were selected from priority districts for the She Conquers Campaign^a^.	Four of the 10 provinces in Zimbabwe were randomly selected: Harare, Manicaland, Mashonaland West, and Midlands.
Facility selection	Sixteen facilities were selected in consultation with the government for the survey, prioritizing facilities already offering PrEP. Facilities where providers had the highest and lowest scores in each county were selected for inclusion in the IDIs, totaling 13 facilities.	*PrEP experienced*: Eight facilities providing PrEP (to sex workers and MSM) and 9 facilities that may provide PrEP in the future (university clinics, public facilities) were purposefully selected for a mix of delivery model (fixed clinic, mobile services, or both), rural/urban, and PrEP uptake rate. *PrEP inexperienced*: Five facilities with sufficient numbers of eligible survey participants.	Facilities providing ART services to > 50 patients; 26 facilities selected.
Participant selection	Survey participants — all PrEP experienced — with highest and lowest 5 % of attitude scores. Additional providers were selected to ensure that 10 providers were selected from each county.	*PrEP experienced*: Aimed to conduct at least 3 IDIs per facility. *PrEP inexperienced*: Highest and lowest 15 % of attitude scores and all counselors and clinicians due to low number of survey participants from these cadres.	Included both PrEP-experienced and -inexperienced providers. Survey participants with highest and lowest 10 % of attitude scores. Also selected all survey participants currently providing PrEP and 3 additional providers who did not participate in the survey to ensure representation of current PrEP providers.

^a^She Conquers is a three-year national campaign launched by the South Africa National Department of Health to reduce HIV infections and improve other health, education, and economic outcomes among AGYW.

Country research teams chose sites and facilities for the in-depth interviews in collaboration with the ministries of health and PrEP technical working groups to ensure the research was responsive to individual country needs. For example, we sampled a mix of sites that were and were not offering PrEP in Zimbabwe because the national Ministry of Health and Child Care (MOHCC) was preparing for PrEP scale-up at the time of the study and was interested in understanding the attitudes of both PrEP-experienced and PrEP-inexperienced providers. Kenya was already providing PrEP nationally; based on the PrEP national research agenda, Ministry of Health (MOH) advisors wanted data from facilities providing PrEP to assess how services were being offered. Details on country-specific site and facility selection are provided in Table 1. Interviewed cadres included doctors, nurses, counselors, and community workers, selected because they have the most direct contact with PrEP clients.

### Data collection

The research conformed to international ethical standards as stated in the United States Federal Policy for the Protection of Human Subjects and was approved by the Amref Health Africa Ethics and Scientific Review Committee in Kenya, the Medical Research Council of Zimbabwe, the Wits Human Research Ethics Committee in South Africa, and the FHI 360 Protection of Human Subjects Committee in the United States. All participants provided written informed consent before participating.

Experienced male and female qualitative data collectors trained in research methods and ethics and sensitized on the study protocol conducted interviews in the participant’s preferred language (English, Swahili, Kamba, or Dholuo in Kenya; English, isiXhosa, or Zulu in South Africa; and English, Shona, or Ndebele in Zimbabwe). The data collectors called survey participants who had agreed to be contacted for potential participation in an IDI and were selected using the extreme case sampling described above. Study participants did not know personal details about the data collectors and had no relationship with them prior to the study. If someone was not reached after three calls or if the survey participant refused to be interviewed, another survey participant who had agreed to be contacted for an IDI and fell within those selected by extreme case sampling was called. Interviews were scheduled with those who agreed to be interviewed. 5 % of participants who were contacted refused to participate due to various reasons, including no longer working at the target facility, refusing to be audio-recorded, being busy with other responsibilities, and being on leave. 12 % of participants could not be reached.

Data collection took place from July to October 2018. Trained interviewers explained the purpose of the interviews, consented participants, and conducted in-person IDIs in private locations convenient to participants where they could not be overheard. Interviews were audio-recorded. Some participants in Zimbabwe did not consent to be audio-recorded due to concerns regarding an upcoming election. For these interviews, data collectors were accompanied by a note-taker and paused frequently to ensure the note-takers were able to document the interviews near-verbatim. Participants in Zimbabwe and Kenya were reimbursed for their time in accordance with local standards.

Data collectors used a semi-structured interview guide (see Additional File 2), available in English and local languages, that covered experiences providing PrEP and other HIV services to AGYW and attitudes about acceptable ages of sexual debut and PrEP use for adolescents. The IDIs explored attitudes separately for adolescent girls and young women. The guide also included a vignette describing a fictional adolescent PrEP client (called “Tshepiso” in South Africa, “Tafadzwa” in Zimbabwe, and “Pendo” in Kenya, but hereafter referred to as “Pendo”), with follow-up questions about the best HIV prevention options for her, whether she should disclose PrEP use to her partner and parents, concerns about her using PrEP, and what support she would need to use PrEP successfully. Vignettes are used in qualitative research to explore participant’s attitudes and beliefs [37].

The interview guides were pilot tested in each country. The research team met weekly during data collection to discuss the status of recruitment and data collection; the data collectors confirmed that data saturation had been reached — meaning no new themes were emerging — when the planned number of interviews had been completed [38].

### Data management and analysis

The data collectors simultaneously transcribed and translated the interviews into English, and then reviewed the transcripts for accuracy. In South Africa, the transcripts were also back translated and reviewed for accuracy by an external translation company. Data were coded and analyzed by the authors — including analysts from in the US and each country — using an applied thematic analysis approach [39]. Coding was conducted in NVivo 11 [40] using a codebook developed a priori based on the interview guide, with additional thematic codes added during the analysis process. The a priori codes were based on the survey results and the interview guide, both of which were informed by the experience of the co-authors’ organizations implementing PrEP services and literature on barriers to adolescents and young people accessing health services. Coding was conducted by country in teams of four to six analysts, who noted key differences by PrEP-experienced versus PrEP-inexperienced.

To assess inter-coder agreement, a team of eight analysts coded selected transcripts independently, compared coding, and resolved differences through discussion. Changes to the coding approach or codebook were documented, and previously coded transcripts were reviewed and updated for consistency. For each country, inter-coder agreement assessments continued until the coding team reached agreement of 80 % or higher [41].

Analysts ran code reports in NVivo Version 11 and reduced and organized the data into themes [36, 39]. After synthesizing the results across the three countries, analysts compared responses to select qualitative questions by providers with positive and negative attitude scores to assess any systematic differences.

## Results

### Description of participants

We conducted 113 IDIs (Kenya = 40, South Africa = 46, and Zimbabwe = 27), which lasted 55 min on average. Participants included 78 providers with experience delivering PrEP and 35 without PrEP experience. The average age was 33 years and the majority (70 %) were female. Experienced PrEP providers primarily provided counseling and education on PrEP. Nearly two-thirds of IDI participants were community-based workers and nurses; 91 % had experience delivering health services to young women and 77 % to adolescent girls (see Table 2).

**Table 2:** Participant demographics

	Kenya (*n* = 40)	South Africa (*n* = 46)	Zimbabwe (*n* = 27)	Overall (*n* = 113)
	n (%)	n (%)	n (%)	n (%)
Age, *mean ± SD*^a^	33.1 ± 8.3	30.0 ± 10.5	38.7 ± 9.9	33.4 ± 10.1
Sex^a^				
Male	13 (33 %)	8 (21 %)	10 (37 %)	31 (30 %)
Female	27 (68 %)	30 (79 %)	17 (63 %)	74 (70 %)
Position^a^				
Clinician	11 (28 %)	2 (5 %)	6 (22 %)	19 (18 %)
Nurse	10 (25 %)	7 (18 %)	12 (44 %)	29 (28 %)
Counselor	9 (23 %)	6 (16 %)	3 (11 %)	18 (17 %)
Community-based worker	10 (25 %)	23 (61 %)	6 (22 %)	39 (37 %)
Self-reported responsibilities related to PrEP (multiple responses possible)^b^				
Prescribing	17 (43 %)	3 (8 %)	7 (29 %)	27 (26 %)
Resupplying	16 (40 %)	3 (8 %)	8 (33 %)	27 (26 %)
Counseling	27 (68 %)	2 (5 %)	13 (54 %)	42 (41 %)
Community Outreach	16 (40 %)	4 (11 %)	8 (33 %)	28 (27 %)
Education	30 (75 %)	7 (18 %)	16 (67 %)	53 (52 %)
None (PrEP inexperienced)	1 (3 %)	28 (74 %)	6 (25 %)	35 (34 %)
Self-reported service delivery experience with (Multiple responses possible)^b^				
Adolescent girls	34 (85 %)	22 (58 %)	23 (96 %)	79 (77 %)
Young women	35 (88 %)	34 (89 %)	24 (100 %)	93 (91 %)
Self-reported familiarity with PrEP for HIV prevention^a^	38 (95 %)	21 (55 %)	23 (85 %)	82 (78 %)
Self-reported receipt of training on oral PrEP?^b^	17 (43 %)	8 (21 %)	8 (33 %)	33 (32 %)

NOTE: some percentages add to more than 100 % due to rounding; ^a^8 missing in South Africa; ^b^8 missing in South Africa, 3 missing in Zimbabwe

### Attitudes toward and perceptions of PrEP for AGYW

By following up on potential barriers to PrEP provision that emerged from the survey results, the qualitative interviews provide a nuanced understanding of providers’ attitudes and perceptions of PrEP for AGYW. Results are summarized by interview topic below, and illustrative quotes are presented in Table 3. The results were similar across countries unless otherwise noted. Only a few minor differences emerged between PrEP-experienced and -inexperienced providers; a few PrEP-inexperienced providers showed a preference for AGYW using condoms over PrEP, and some shared concerns with AGYW ability to take PrEP daily. Similarities and differences between participants with positive and negative attitude scores from the survey are detailed at the end of this section.

**Table 3:** Illustrative quotes

**Acceptable age for sexual activity and PrEP use**	*They are still young…this is when they are still in high school … where you concentrate. It shapes your life from there, so sex can wait.* – Nurse, Kenya, previously provided PrEP *It’s inappropriate for adolescents between age 15 and 18 to engage in sexual intercourse…they shouldn’t take PrEP either. But if they feel […] they are wise enough to engage in such thing, then it’s okay. […] It’s their choice. I mean they have rights but with these rights come responsibilities.* – Community worker, South Africa, not providing PrEP *As a mother, if I see a girl of 17 years taking PrEP, I wouldn’t like it. But that is the reality of what’s happening with these girls today. As a health worker I have seen PrEP helping others, so it’s rather better for her to take the PrEP.* – Nurse, Zimbabwe, providing PrEP
**Best HIV prevention options**	*For me, I would go for PrEP because like I said, women have low bargaining power. […] You know she cannot tell the boyfriend to use a condom.* – Clinician, Kenya, not providing PrEP *She should use both oral PrEP and at the same time using protection [condoms] [...] At least she will have a backup of oral PrEP. Sometimes maybe they can use condoms, [but] at the same time the guy might refuse to use condoms. At least at that time she will be using oral PrEP.* – Nurse, Zimbabwe, not providing PrEP
**Concerns about PrEP**	*She won’t be able to take it correctly because she’s afraid of being found out by [her partner]. […] Maybe he could react negatively and beat her or ask why she didn’t tell him that she is using this prevention.* – Community worker, South Africa, not providing PrEP *She will stop [using condoms]. […] She will say “I’m protected. I can’t get HIV” […] she knows she is protected she can’t get HIV so there are some risky behaviors […] I will [still offer her PrEP] because she is willing to take them [PrEP] home. I think everybody knows his or herself. I know she will find a way of how to take them despite the husband knowing.* – Counselor, Kenya, providing PrEP
**Male partners**	*I usually leave [disclosure] at her discretion, if she wants to its okay and if she does not want to, it’s still okay […] it’s at her comfort because she is the one who knows the boyfriend. […] I would just tell her first try and ask him if he is willing to go for HIV test. If you see the reaction is bad, that means if you start telling him about the PrEP it will also bring more issues to it, so she would rather keep it to herself.* – Nurse, Kenya, providing PrEP *[Her partner] is not the person to talk to […] he will react negatively and also he may even get violent because now at the moment she strongly suspects him of having multiple partners. If she says that she is taking PrEP because of that, maybe they may get into a fight, so I don’t think that she should tell him.* – Community worker, South Africa, not providing PrEP
**Parents**	*Yes, at her age she should tell her parents that she is using oral PrEP. She must inform them so that she is able to take them consistently, and she can also experience side effects and they will not know how best to help her.* – Community worker, Zimbabwe, not providing PrEP *At times someone can come and pick PrEP but later call to inform me, “my mother threw away the drugs. Maybe she doesn’t understand what kind of drugs they are.”* – Community educator, Kenya, providing PrEP
**Support needed**	*Whoever will be administering [PrEP] to her just to check on her least once a month or so [to see] if she takes her pills regularly and how she is doing, if she has side effects or anything. I think she needs an emotional support.* – Community worker, South Africa, not providing PrEP *[She will need] A lot of counseling and advice. And in that particular moment before she starts, I will even try to engage the partner… to talk on behalf of her on the importance of taking this [PrEP] and why it is not a crime on taking them…* – Clinician, Kenya, providing PrEP *[Pendo] needs adherence counseling and side effects management, appointment reminders and relationship counseling would be necessary too […]I would also tell her that PrEP does not protect her from pregnancy and STIs.* – Counselor, Zimbabwe, not providing PrEP
**Service delivery to AG versus YW**	*[Women ages] 20-24 years, I have seen that their [PrEP] retention rate is a little better in comparison to those below 20. Yes, I think they more focused, they know their problems, they know their risk, and they are determined.* – Nurse, Zimbabwe, providing PrEP *At least at that age 20-24 [years] someone has started to mature even when you give them information […] they show up, they listen. You know at around 20 years, someone already knows what they want to do with their lives so they can't let you down.* – Community worker, Kenya, providing PrEP
**Uptake, adherence, retention challenges**	*They [AG] are afraid of their parents, they are afraid of the community, they are afraid of going to access these services because they feel that they are judged by the providers […] with that fear comes the denial that they are sexually active.* – 22-year-old female community educator, South Africa, does not provide PrEP *They are worried about being caught taking [PrEP] tablets cause it’s not known in the community. Their boyfriends do not know about it, so if they see their tablets they will think that they are positive.* – Clinician, Zimbabwe, providing PrEP *The challenge is just about stigma […] If an adolescent girl comes who is HIV-negative and wants PrEP … the next question is how would my parents take me because the drugs are stored at home, so once the parent sees the bottle maybe the parent can have a notion that this girl maybe she is behaving [way] that is not pleasing to the parent … to the society she is taking PrEP because her behavior is not good.* – Clinician, Kenya, providing PrEP
**Strategies used to address challenges**	*There are times when I go beyond and give the adolescents my WhatsApp phone number so that they may get in touch if they have a problem. It is because they do not have anyone to talk to. It becomes easier for them to be in touch if they have a problem after we create rapport. We can be in contact by phone before things escalate.* – Nurse, Zimbabwe, not providing PrEP *I think creating awareness and letting them know that it’s actually okay to come to those clinics and actually talking to not only them but the communities to know that because HIV is not only an adolescents’ issue but a community issue. To make people aware that this is just another way to prevent this disease from spreading even wider.* – Community educator, South Africa, not providing PrEP *We exchange phone numbers so we can talk. When we realize they have missed appointments I say, “You were supposed to come today.” So I make a follow up using WhatsApp, and if I can’t get hold of her I ask the peer counselors to make a follow up.* – Nurse, Zimbabwe, providing PrEP

#### Overall perception of PrEP for AGYW

Across the topics that were explored in IDIs, one-third of providers voiced one or more concerns or hesitations, but few expressed consistently negative attitudes or serious concerns throughout the interview. For example, a PrEP-experienced clinician in Kenya stated that PrEP was a good option for “Pendo,” the 17-year-old girl in the vignette, because, “*she has fears that the boyfriend has several sexual partners*,” but the clinician recommended that she first try to abstain from sex because “*she is an adolescent. That means she can wait to get to the mature age.”* When asked about offering Pendo PrEP, the clinician replied, “*For now no, because you know these drugs are meant for preventive measures and they have to be used as prescribed. So, if there is any non-adherence issue, she is at risk of getting HIV.”* However, the clinician also stated that they would be comfortable offering PrEP to Pendo if she were someone they knew: “*I am not concerned about what the lady is using, my concern is about what she might get…we just want to prevent […] HIV*.”

#### Acceptable age for sexual activity and PrEP use

Many providers said girls engaged in sexual activity at an earlier age than they thought was appropriate. The majority preferred that girls wait until they are 18. The reasons providers cited for this preference included that 18 is the age when girls typically complete secondary school, the legal age of consent in Zimbabwe and Kenya, and the age when young people become “more mature.” A few — mostly PrEP-inexperienced providers — felt strongly that ages 15 to 19 were “too young” for sex and that “sex could wait.”

Despite their concerns about adolescent girls being sexually active, IDI participants acknowledged that many adolescent girls need HIV prevention education, and those at risk of HIV could benefit from PrEP. Most IDI participants said that it is acceptable for AGYW to initiate PrEP when they are 15 to 19 years old or as soon as they are sexually active. Only a small minority of providers said 15 to 19 was “too young” to initiate PrEP, because they are “children” and should “study first.”

#### Best HIV prevention options

When providers were asked what they thought were the best HIV prevention options for Pendo, condoms and PrEP were mentioned most frequently, though few talked about using them simultaneously. They discussed the relative advantages and disadvantages of these methods, acknowledging that neither is perfect. They noted that condoms have the advantage of preventing HIV, other STIs, and pregnancy, while PrEP prevents only HIV. Condoms were also viewed as easier for young people to access and use. Providers said the main advantage of PrEP for Pendo was that she could use it without the knowledge or consent of her partner, in contrast to condoms, which would likely be challenging for her to negotiate. However, many acknowledged that PrEP use might be difficult to conceal from a partner, who might react negatively to its use. When asked which method they would recommend Pendo try first, most said condoms, because they provide protection against other STIs and pregnancy as well as HIV. In Zimbabwe, some responses varied by PrEP experience. A few providers, most of whom were PrEP-experienced, recommended that she try PrEP in conjunction with condoms, while the majority, who were largely PrEP-inexperienced, recommended she try condoms alone.

#### Concerns about PrEP

Participants’ primary concern with Pendo using PrEP was whether she would be able to adhere to daily use, especially if her partner or parents disapproved or if she was trying to use it without their knowledge. A small minority of providers were concerned about the potential for decreased condom use and increased risky behavior. They reasoned that once Pendo was on PrEP, “there is nothing she will be worried about.” They also pointed out that her partner might pressure her not to use condoms.

Despite their concerns, most providers said they would offer PrEP to Pendo because of her risk of HIV and potential difficulty negotiating the use of other HIV prevention methods. They said they “want her to be safe,” and that it is better to take PrEP than “leaving things to chance.” A few providers — mostly PrEP-inexperienced — were opposed to offering Pendo PrEP, primarily because of concerns about her ability to adhere to daily use.

Participants were asked if their responses would change if Pendo were someone they knew. Most said they would still provide PrEP to someone they knew, saying they would have “no problem” or would be “very happy,” because it would be better for her to use PrEP than to be at risk of HIV. Others had concerns about her being sexually active at “such an early age” and acknowledged the disconnect between how they felt as health providers and how they felt as parents.

#### Male partners

Participants said that the decision of whether to disclose PrEP use to a male partner was complex. Those who thought Pendo should disclose PrEP use to her partner said disclosure could facilitate adherence and continuation. She would not have to worry about hiding her PrEP use, and her partner could remind her to take PrEP daily or help her get refills. Others stated that disclosure was important to ensuring trust and transparency in her relationship. Many providers, especially in Kenya, stated that disclosure might encourage her partner to get tested or start using PrEP himself.

Several participants thought Pendo’s decision should be based on how much negotiating power she has in the relationship and how she thinks her partner will react, particularly whether he might react violently. Participants reasoned he might misunderstand her PrEP use to indicate that she does not trust him, has other partners, or is HIV-positive. Some providers in South Africa viewed PrEP use as an individual decision more than a relational one, calling it “a personal thing” that her partner “doesn’t have to know about,” and saying that “it’s her life.” Providers recommended that Pendo gauge her partner’s potential reaction by bringing up HIV testing and prevention generally, or that she use partner and/or couples’ HIV counseling and testing services to facilitate disclosure of PrEP use.

#### Parents

Participants were asked about Pendo disclosing PrEP use to parents. One-third to one-half of participants in each country said Pendo should disclose to her parents because they could provide encouragement and could support her adhering to daily use, obtaining PrEP refills, and managing side effects. Those who thought she should not disclose to her parents said there was “no need” or thought her parents would not react well. Disclosure would reveal that Pendo is sexually active, which her parents might not approve of; some even said that telling her parents would “destroy the relationship.” They also said parents may not understand what PrEP is because of low community awareness about it, including mistaking PrEP for ART. Even providers who thought Pendo *should* disclose noted that disclosure could be challenging for these same reasons.

Some providers said Pendo should decide whether to disclose based on the nature of her relationship with her parents. For example, if her parents were “modern,” “open-minded,” or “understanding,” she could talk to them about her PrEP use, but if they were “strict” it would be better not to discuss PrEP with them. A few providers in Zimbabwe had specific recommendations, such as suggesting that Pendo talk to her mother more generally about PrEP first, to gauge her reaction, or involve a counselor in the discussion.

#### Differences between participants with positive and negative attitudinal scores

For some IDI questions, participants’ responses aligned with their categorization of having positive or negative scores from the survey. For example, providers with positive attitudes from the survey tended to show more accepting attitudes in the IDIs regarding initiating PrEP at younger ages compared to those with negative scores who more often reported in IDIs that they thought adolescent girls should wait until they are 18 or older to have sex. Providers with negative attitudes from the survey most often recommended in IDIs that Pendo try condoms and abstinence or end the relationship. Providers with positive attitudes from the survey more often expressed that PrEP was a good option for Pendo.

For other IDI questions, there were no differences between providers with positive and negative attitude scores from the survey. Both extremes shared similar types of concerns about PrEP, and they expressed similar opinions about offering PrEP to someone they knew.

### Experiences delivering PrEP and other HIV services to AGYW

#### Service delivery to AG versus YW

Reflecting on their experiences providing PrEP and other HIV services, providers across the three countries reported that they faced more challenges delivering services to adolescent girls compared with young women. They characterised adolescent girls as “troublesome,” “not listening,” “reluctant” to seek services or receive information, “clueless” about their partners’ behavior and “dishonest.” By comparison, they described young women as “more mature,” “focused,” willing to talk about sex and to “listen more,” and knowing how to “take care of themselves.”

These perceptions were attributed to the difference in life stages. Providers noted that young women live on their own or are married with children, which gives them more freedom to seek services and increased motivation to continue taking PrEP, whereas adolescents are more likely to live at home and be in school, which poses challenges of parental involvement and reduced mobility and access to services. Providers also said age differences can make it challenging for them to relate to adolescent girls. They suggested that adolescent girls may feel more comfortable discussing sensitive topics with peers or younger health workers.

#### Uptake, adherence, and continuation challenges

IDI participants with experience providing PrEP confirmed that uptake, adherence, and continuation among AGYW have been challenging, particularly for adolescent girls. They cited lack of PrEP awareness, stigma, and lack of PrEP disclosure to partners and parents as the primary barriers. Some also mentioned side effects as a barrier to PrEP use.

PrEP-experienced providers said PrEP awareness is low among AGYW and communities; most notable is the lack of understanding about the differences among PrEP, post-exposure prophylaxis (PEP), and ART. They also talked about the difficulties AGYW experienced using PrEP without telling their partners and parents. Lack of awareness about PrEP in the community and fear of negative reactions were cited as the primary reasons for not disclosing PrEP use to partners and parents. Providers reported that some parents received specific information about PrEP that helped garner their support and were then able to facilitate their daughters’ PrEP adherence.

Providers talked about two types of stigma as barriers to PrEP uptake and use: HIV stigma and stigma related to being sexually active. They said AGYW are concerned that community members who see a young woman access PrEP service will assume she is taking antiretroviral pills because she is HIV-positive. They also worry about parents or partners discovering the tablets or hearing them rattle in the bottle. Providers explained that adolescents feared being stigmatized if they used PrEP or accessed other HIV services, because that would mark them as someone who is sexually active, and they would be labeled “promiscuous” or assumed to be engaged in sex work. Providers said AGYW clients did not report experiencing stigma related to PrEP use, but their fear of being stigmatized was an important influence on their service-seeking behavior. Providers discussed HIV stigma as an issue that affected adolescent girls more than young women.

#### Service delivery strategies

PrEP providers shared strategies they were using to facilitate PrEP uptake, adherence, and continuation, including intensified counseling to address adherence and continuation challenges; community discussions to address stigma and normalize PrEP for AGYW; and peer support groups to encourage adolescent girls to share their challenges. They also track missed client visits and follow up with calls, SMS, and home visits to promote continuation. Providers discussed strategies used in other HIV services that could be applied to PrEP, including using social media and digital platforms to reach AGYW; supporting AGYW‒parent relationships through counseling, assisted disclosure, and health talks with parents; encouraging couples’ HIV testing and counseling; structuring services to improve anonymity and confidentiality; and providing services at places and times convenient for students.

## Discussion

Providers in this study were generally supportive of PrEP for AGYW, regardless of their experience providing PrEP. They had more reservations about PrEP use among adolescent girls compared to young women, rooted in (1) negative attitudes about adolescent girls being sexually active and (2) concern about the ability of AG to adhere to daily use, particularly when using PrEP without disclosing to parents and partners. These concerns are consistent with other studies of provider attitudes toward PrEP use among adolescent girls [27, 31]. A study with community gatekeepers in South Africa before PrEP was rolled out to AGYW found “moral anxiety about youth sexuality” as a main theme, and health providers talked about their role in “guardianship of adolescent sexuality” [32].

Participant concerns about adherence are supported by data from trials and national programs, which show that younger age is associated with lower adherence and continuation [42, 43]. PrEP-experienced providers in the study suggested that AGYW could be supported to use PrEP effectively through peer support groups, social media and digital platforms, and relationship counseling with parents and partners. These and other promising approaches — weekly communication from providers, integration of PrEP delivery into comprehensive sexual health programs, and cash incentives [42, 44–48] — should be evaluated with AGYW for effectiveness and feasibility.

Despite the concerns and challenges that providers in this study identified about delivering PrEP to adolescent girls, most acknowledged that adolescent girls could benefit from PrEP because of their risk of HIV and inability to negotiate condoms, including participants with negative attitude scores based on their responses to the quantitative survey. Further, most said they would be willing to offer PrEP to adolescent girls. Providers’ stated recognition of AG’s need for additional HIV prevention methods and their willingness to provide it are encouraging, though additional research on whether and how this willingness translates to actual service provision would be useful.

Consistent with other studies [27, 32, 44, 45, 49–54], participants reported that the main barriers to PrEP uptake and use among AGYW were lack of PrEP awareness among AGYW and communities; fear of disclosing PrEP use to partners and parents; and stigma related to HIV and to adolescent sexuality. These results suggest that intensive community sensitization to promote PrEP awareness, combat stigma, and address social norms around adolescent sexuality [32, 42, 51, 52, 55] is critical to making PrEP a viable HIV prevention option for AGYW and other vulnerable populations. Specific messages and events for parents, male partners, and community leaders could promote awareness, provide accurate information about PrEP, and help AGYW who want to use PrEP gain the support of their parents and partners. At the same time, building the skills and efficacy of stigmatized groups to challenge stigma has been identified as a best practice [56]; training AGYW to be PrEP ambassadors in their communities could help reduce PrEP stigma and promote AGYW PrEP use [57]. Finally, promoting PrEP as a method for anyone who wants to promote wellness, rather than a method for high-risk — and often highly stigmatized — groups, could help reduce stigma [45, 58–60].

### Research utilization

Findings from this study have influenced provider training in the study countries. OPTIONS developed an AGYW Provider Training Package that included values clarification exercises to address specific negative attitudes revealed in the study and promote youth- and adolescent-friendly PrEP services [61]. These values clarification exercises were incorporated by the MOHCC in Zimbabwe into national PrEP provider trainings. In Kenya, the National PrEP Curriculum was updated to include the vignette used in this study. In South Africa, study findings informed the revision of the national PrEP service provider training to address emerging concerns regarding PrEP provision to adolescent girls.

Study results also informed the development of an HIV Prevention Ambassador Training Curriculum for Adolescent Girls and Young Women [62], which prepares AGYW ambassadors to support their peers to learn about PrEP as part of combination HIV prevention and to support peers who decide to use PrEP. To address the barriers to AGYW PrEP use that providers raised in this study, we included tools to help AGYW develop skills to talk with their parents and partners about PrEP and address community misconceptions about PrEP. The curriculum has been used to train ambassadors in Kenya, South Africa, and Zimbabwe, and these ambassadors have supported PrEP community awareness campaigns in Chitungwiza, Zimbabwe, and Kiambu County, Kenya.

### Limitations

Providers’ attitudes about PrEP for AGYW were self-reported and may not be entirely accurate given the likelihood of implicit bias. Barriers to PrEP uptake, adherence, and continuation were reported from the point of view of providers and may not fully reflect clients’ experiences. In addition, data collection occurred when the three countries were in the early stages of national PrEP rollout; it is possible provider perspectives have evolved with experience and as additional provider training and community awareness campaigns have been conducted.

Another limitation of this study is that the samples differ by country, because the sampling approach for each country was designed to be directly responsive to in-country needs and priorities and was not designed for forming cross-country comparisons. During country-specific analyses, similar themes emerged across countries, leading to the decision to report the multi-country findings together. Although we noted results that emerged from a specific country, we were unable to systematically compare results across countries because of the difference in samples by country. Nonetheless, the convergent results point to important issues that could be addressed to improve PrEP service delivery for AGYW. These results could be used to inform future research that could investigate differences in providers’ attitudes based on provider age, PrEP experience, cadre, or gender or explore to what extent provider attitudes were shaped by country-level differences in culture, policy, or PrEP rollout strategy.

## Conclusions

In summary, providers who participated in this research were generally accepting of PrEP as an HIV prevention option for adolescent girls and young women though many had negative attitudes about adolescent girls being sexually active. They also had concerns about whether adolescent girls could take PrEP daily, particularly given the lack of PrEP awareness in the community and the challenges of disclosing PrEP use to parents and partners. To promote effective delivery of PrEP for AGYW, national PrEP provider training in Kenya, South Africa, and Zimbabwe was updated to address negative attitudes about adolescent sexual activity and concerns about PrEP. In addition, a training curriculum for AGYW HIV prevention ambassadors was created to address potential barriers to PrEP use identified in this study. These findings could inform the introduction and scale-up of PrEP for AGYW in other countries and potential future introduction of new ARV-based HIV prevention methods such as the dapivirine ring and injectable PrEP.

## Supplementary Information

**Additional file 1:** Survey responses about PrEP for AGYW by country. Survey responses to attitudinal questions about acceptability of PrEP for AGYW, reported by country 

**Additional file 2:** Interview Guide Questions about PrEP for AGYW. Interview questions developed for this study to assess providers’ attitudes about PrEP for AGYW and experiences delivering PrEP to AGYW 

## Data Availability

The datasets generated and/or analyzed during the current study are not publicly available due to potential sensitivity of the content but are available from the corresponding author on reasonable request.

## Abbreviations

AGYW: Adolescent girls and young women
ART: Antiretroviral therapy
GBV: Gender-based violence
MSM: Men who have sex with men
MOHCC: Ministry of Health and Child Care
NGO: Nongovernmental organization
OPTIONS: Optimizing Prevention Technology Introduction On Schedule
PEP: Post-exposure prophylaxis
PrEP: Pre-exposure prophylaxis
PEPFAR: President's Emergency Plan for AIDS Relief
STIs: Sexually transmitted infections
USAID: U.S. Agency for International Development

## Footnotes

1 In Kenya, “adolescent girls” were defined as ages 15‒19 and “young women” as 20‒24, per national standards.
