# Supplementary material for: Health care providers’ attitudes toward and experiences delivering oral PrEP to adolescent girls and young women in Kenya, South Africa, and Zimbabwe
Source: BMC Health Serv Res. 2021 Oct 18;21:1112. doi: 10.1186/s12913-021-06978-0 (PMC8522219; doi:10.1186/s12913-021-06978-0)
Supplement: Supplementary file 1 — Additional file 1: Survey responses about PrEP for AGYW by country. Survey responses to attitudinal questions about acceptability of PrEP for AGYW, reported by country [file 12913_2021_6978_MOESM1_ESM.pdf]

SOUTH AFRICA

	STATEMENT	AGE GROUP	Strongly Agree	Agree	Neutral	Disagree	Strongly Disagree
**Disagreement** indicates more favorable attitude/ perception	PrEP should not be given to AG/YW because they should not be having sex	AG	8%	9%	10%	57%	15%
		YW	3%	8%	7%	71%	10%
	It is better to tell sexually active unmarried AG/YW to abstain from sex rather than give her PrEP	AG	25%	24%	15%	31%	5%
		YW	12%	22%	12%	52%	3%
	Unmarried adolescents should get parental consent to use PrEP	AG	11%	25%	15%	41%	8%
		YW	-				
	Providing PrEP to unmarried AG/YW promotes sexual promiscuity	AG	12%	32%	20%	31%	4%
		YW	7%	32%	15%	40%	5%
	Providing PrEP to AG/YW would result in backlash from the community	AG	9%	34%	29%	25%	3%
		YW	5%	27%	25%	41%	2%
	An AG/YW should tell her partner if she is using PrEP	AG	12%	24%	23%	36%	6%
		YW	6%	39%	21%	30%	5%
**Agreement** indicates more favorable attitude/ perception	PrEP should be available to all AG/YW	AG	35%	41%	15%	7%	2%
		YW	34%	61%	3%	2%	
	AG/YW are responsible enough to be able to use PrEP consistently	AG	9%	38%	23%	26%	4%
		YW	14%	72%	8%	4%	1%
	Health workers should provide PrEP to both married and unmarried AG/YW	AG	30%	54%	10%	4%	2%
		YW	25%	70%	3%	2%	
	Information on PrEP should be part of HIV prevention education for AG/YW	AG	46%	50%			
		YW	39%	60%			
	AG/YW should be comfortable to ask their provider for PrEP	AG	21%	71%	5%	3%	
		YW	26%	74%	1%		
	Parents should be comfortable if their adolescent daughters use PrEP	AG	12%	47%	19%	19%	3%
		YW					

Some rows total 99% or >100% due to rounding.

ZIMBABWE

Disagreement indicates more favorable attitude/ perception

STATEMENT	AGE GROUP	Strongly Agree	Agree	Neutral	Disagree	Strongly Disagree
PrEP should not be given to AG/YW because they should not be having sex	AG	6%	7%	8%	60%	19%
	YW	3%	4%	6%	67%	20%
It is better to tell sexually active unmarried AG/YW to abstain from sex rather than give her PrEP	AG	24%	20%	10%	42%	4%
	YW	13%	19%	9%	47%	12%
Unmarried adolescents should get parental consent to use PrEP	AG	13%	20%	12%	43%	13%
	YW	-				
Providing PrEP to unmarried AG/YW promotes sexual promiscuity	AG	17%	33%	13%	31%	5%
	YW	7%	35%	9%	42%	8%
Providing PrEP to AG/YW would result in backlash from the community	AG	20%	50%	8%	19%	4%
	YW	8%	35%	17%	37%	3%
An AG/YW should tell her partner if she is using PrEP	AG	21%	31%	20%	23%	5%
	YW	16%	37%	22%	22%	3%

Agreement indicates more favorable attitude/ perception

STATEMENT	AGE GROUP	Strongly Agree	Agree	Neutral	Disagree	Strongly Disagree
PrEP should be available to all AG/YW	AG	35%	28%	9%	21%	6%
	YW	49%	35%	5%	10%	1%
AG/YW are responsible enough to be able to use PrEP consistently	AG	7%	38%	20%	31%	4%
	YW	23%	49%	11%	15%	
Health workers should provide PrEP to both married and unmarried AG/YW	AG	28%	55%	6%	8%	4%
	YW	35%	54%	6%	5%	
Information on PrEP should be part of HIV prevention education for AG/YW	AG	63%	34%			
	YW	57%	41%			
AG/YW should be comfortable to ask their provider for PrEP	AG	39%	53%	3%	5%	
	YW	52%	46%	1%	1%	
Parents should be comfortable if their adolescent daughters use PrEP	AG	16%	47%	22%	10%	5%
	YW					

Some rows total 99% or >100% due to rounding.

KENYA

Statement	Age group	Strongly Agree	Agree	Neutral	Disagree	Strongly Disagree
**Disagreement** indicates more favorable attitude/perception						
PrEP should not be given to AG/YW because they should not be having sex	AG	4%	4%	8%	47%	37%
	YW	3%	2%	5%	47%	43%
It is better to tell sexually active unmarried AG/YW to abstain from sex rather than give her PrEP	AG	28%	23%	17%	23%	9%
	YW	17%	22%	13%	29%	20%
Unmarried adolescents should get parental consent to use PrEP	AG	12%	22%	13%	40%	13%
	YW	-				
Providing PrEP to unmarried AG/YW promotes sexual promiscuity	AG	15%	31%	15%	28%	10%
	YW	13%	26%	12%	36%	18%
Providing PrEP to AG/YW would result in backlash from the community	AG	13%	36%	17%	24%	9%
	YW	8%	30%	14%	37%	12%
An AG/YW should tell her partner if she is using PrEP	AG	31%	32%	17%	17%	3%
	YW	27%	40%	14%	14%	4%
**Agreement** indicates more favorable attitude/perception						
PrEP should be available to all AG/YW	AG	25%	25%	14%	26%	10%
	YW	36%	30%	10%	17%	4%
AG/YW are responsible enough to be able to use PrEP consistently	AG	19%	33%	17%	23%	8%
	YW	27%	41%	12%	14%	5%
	AG	28%	40%	8%	18%	6%
	YW	37%	42%	6%	11%	4%
	AG	64%	31%	2%		2%
	YW	63%	33%	1%	1%	2%
AG/YW should be comfortable to ask their provider for PrEP	AG	39%	48%	4%	6%	2%
	YW	46%	46%	4%	3%	1%
Parents should be comfortable if their adolescent daughters use PrEP	AG	26%	34%	18%	15%	7%
	YW					

Some rows total 99% or >100% due to rounding.
