# Supplementary material for: Health care providers’ attitudes toward and experiences delivering oral PrEP to adolescent girls and young women in Kenya, South Africa, and Zimbabwe
Source: BMC Health Serv Res. 2021 Oct 18;21:1112. doi: 10.1186/s12913-021-06978-0 (PMC8522219; doi:10.1186/s12913-021-06978-0)
Supplement: Supplementary file 2 — Additional file 2: Interview Guide Questions about PrEP for AGYW. Interview questions developed for this study to assess providers’ attitudes about PrEP for AGYW and experiences delivering PrEP to AGYW [file 12913_2021_6978_MOESM2_ESM.doc]

**Additional File 2. Interview Guide Questions about PrEP for AGYW**

**BACKGROUND**

1. To start, please tell me your role at [name of facility] and how long you have worked here.
2. What services do you currently provide?
   1. [probe]: HIV testing, HIV treatment, HIV prevention counselling, family planning,

*Interviewer: Now we will discuss different target populations for oral PrEP. I’ll start by asking you some questions about adolescent girls and young women, ages 15-24.*

**AGYW**

1. From what age do you feel it is okay for adolescents (15-19) to engage in sex if they want to?
   1. Do you feel the same age applies for both boys and girls? Why?
2. From what age do you think it is okay for adolescents (15-19) to begin taking oral PrEP? Why?
   1. Do you feel the same age applies for both boys and girls? Why?
3. [PrEP experienced] What experience do you have in providing oral PrEP services to adolescent girls (19 years or younger)?
   1. What have been the challenges in PrEP service provision to adolescent girls?
   2. What have you done to address those challenges?
4. [PrEP experienced] What experience do you have in providing oral PrEP services to young women (20 – 24 years)?
   1. What have been the challenges in PrEP service provision to young women?
   2. What have you done to address those challenges?

*Interviewer: Now I’d like to give you some examples of different kinds of clients you may talk with to see what you think about their situation.*

Vignette

Pendo, 17 years old, wants to use oral PrEP but is too scared to tell her boyfriend, Baraka, as she fears he will react negatively. She is also nervous about her parents finding out, as she still lives at home. She strongly suspects that Baraka has other partners. Baraka is much older than Pendo. He is 35 years old and gives her money for clothes and airtime. He monitors her very closely and gets angry when she hangs out with other men or goes somewhere without telling him.

- What do you think about [name]’s relationship?
- What do you think is [name]’s level of HIV risk?
- Which HIV prevention options would you suggest for [name]? Why? [behavior change, abstinence, condoms, partner reduction]
- Do you think PrEP would be a good HIV prevention option for [name]? Why?
  - What prevention option do you think she should try first? Why?
- What concerns do you have about [name] taking PrEP?
  - [Probe: Adherence, decreasing condom use, increasing risky sexual behavior, intimate partner violence]
- Would you offer [name] oral PrEP despite your concerns?
- If [name] starts using PrEP, do you feel she should inform her partner? Why or why not?
- If [name] starts using PrEP, do you feel she should let her parents know? Why or why not?
- What kind of support do you think [name] will need if she decides to take PrEP? [adherence, side effect management, appointment reminders, transport or access, relationship counselling]
  - Which of the support areas you have mentioned will you be comfortable to assist in as a health worker?
  - Probe: Why/ Why not
- How would you feel about [name] taking oral PrEP if she was someone you knew (for example, your friend’s daughter)?
